# Unique synapomorphies and high diversity in South American Raji-related Epstein-Barr virus genomes

**DOI:** 10.1590/0074-02760230122

**Published:** 2023-11-03

**Authors:** Paula Alves, Vanessa Emmel, Gustavo Stefanoff, Flavia Krsticevic, Joaquín Ezpeleta, Javier Murillo, Elizabeth Tapia, Edson Delatorre, Eliana Abdelhay, Rocio Hassan

**Affiliations:** 1 Instituto Nacional de Câncer, Centro de Transplante de Medula Óssea, Rio de Janeiro, RJ, Brasil; 2 Instituto Nacional de Câncer, Coordenação de Pesquisa Clínica, Rio de Janeiro, RJ, Brasil; 3 Universidad Nacional de Rosario, Centro Internacional Franco Argentino de Ciencias de la Información y de Sistemas, Rosario, Argentina; 4 Universidade Federal do Espírito Santo, Centro de Ciências da Saúde, Departamento de Patologia, Laboratório de Genômica e Ecologia Viral, Vitória, ES, Brasil

**Keywords:** Epstein-Barr virus, oncovirus, genome, diversity, South America

## Abstract

**BACKGROUND:**

Epstein-Barr virus (EBV) is a human gammaherpesvirus etiologically linked to several benign and malignant diseases. EBV-associated malignancies exhibit an unusual global distribution that might be partly attributed to virus and host genetic backgrounds.

**OBJECTIVES:**

To assemble a new genome of EBV (CEMO3) from a paediatric Burkitt’s lymphoma from Rio de Janeiro State (Southeast Brazil). In addition, to perform global phylogenetic analysis using complete EBV genomes, including CEMO3, and investigate the genetic relationship of some South American (SA) genomes through EBV subgenomic targets.

**METHODS:**

CEMO3 was sequenced through next generation sequencing and its coverage and gaps were corrected through the Sanger method. CEMO3 and 67 EBV genomes representing diverse geographic regions were evaluated through maximum likelihood phylogenetic analysis. Further, the polymorphism of subgenomic regions of some SA EBV genomes were assessed.

**FINDINGS:**

The whole bulk tumour sequencing yielded 23,217 reads related to EBV, which 172,713 base pairs of the newly EBV genome CEMO3 was assembled. The CEMO3 and most SA EBV genomes clustered within the SA subclade closely related to the African Raji strain, forming the South American/Raji clade. Notably, these Raji-related genomes exhibit significant genetic diversity, characterised by distinctive synapomorphies at some gene levels absent in the original Raji strain.

**CONCLUSION:**

The CEMO3 represents a new South American EBV genome assembled. Albeit the majority of EBV genomes from SA are Raji-related, it harbours a high diversity different from the original Raji strain.

Epstein-Barr virus (EBV), also known as human gammaherpesvirus 4 (or HHV-4), is ubiquitous and causally associated with benign and malignant diseases.^1^ EBV-associated neoplasms have an unusual distribution, which may depend on ethnographic and genetic factors of the host and the virus.^2^ ^,^ ^3^ ^,^ ^4^ ^,^ ^5^ Understanding EBV-mediated oncogenesis is an ongoing goal, and attention has been focused on EBV genomic diversity under the hypothesis that genetic polymorphisms may affect cell transformation properties.^6^ ^,^ ^7^ EBV virions have a linear double-stranded DNA genome of approximately 170 kilobase pairs and harbour more than 90 open reading frames and 46 functional small untranslated RNAs, which act concordantly during the latent and lytic cycles.^1^ EBV diversity is mainly based on differences in the EBNA latent gene family, dividing EBV into two major types: EBV types 1 and 2.^6^ Although EBV types are not associated with diseases *per se*, the haplotypic association of type 1 and V3 variants has previously been related to several EBV-associated malignancies.^8^ V3 polymorphisms in the promoter zone (Zp) of the transactivator *BZLF1* lytic gene are responsible for switching from a latent to lytic EBV cycle. More recently, the set of polymorphisms type 1 + V3 were associated to functional gain through the enhance lytic replication.^8^ Despite this important role of the Zp promoter variation, its gene *BZLF1*, has shown to present a low nucleotide variation when compared to latent genes, but despite that, its variants seem to have a probable linkage disequilibrium with its Zp promoter with the *EBNA3C* gene, due to its proximity in the genome.^9^ Due to an extensive and heterogeneous genome and the fact that latent persistence occurs in general at low burden,^1^ few EBV genomes were known until recently.^10^ Hence, attention has been focused on the diversity of subgenomic regions to discern viral factors that are geographically restricted from those associated with specific tumorigenic processes.^10^ ^-^ ^22^ Diverse latent genes demonstrated to be expressed mainly in tumours,^23^ ^,^ ^24^ ^,^ ^25^ ^,^ ^26^ later, it was demonstrated that these genes have an increased number of polymorphisms compared to lytic genes.^26^ From the latent genes, some EBNA family genes had a greater ratio of nonsynonymous to synonymous codon changes, which led to suggesting that they are evolving under positive selection.^26^ EBNA-1 is a 641 amino acid protein, which is essential for replication and persistence of EBV genome in latently infected memory B cells.^27^ Several studies of genetic variation of *EBNA1* demonstrated evidence for a geographic restriction rather than disease association, in addition to show that geographic areas may have different EBNA-1 variants within their host population.^26^ ^,^ ^27^

Another latent gene extensively studied was the latent membrane protein 1 (*LMP1*), and early efforts led to description of the variant CAO harbouring polymorphisms, such as a 30 base pair (bp) deletion (del30) and a 15 bp insertion, related to inducing tumorigenesis *in vivo*.^28^ LMP1 is an EBV latent protein that is highly variable and reflects human migration over the past few centuries.^16^ ^,^ ^18^ ^,^ ^21^ Nonetheless, *LMP1* has the ability to transform and immortalize B cells and epithelial cells *in vitro*, and its polymorphism could be associated with enhanced tumorigenesis capacity.^23^ ^,^ ^28^ Currently, LMP-1 is a well-characterised EBV protein, and several polymorphisms and mutational hotspots have been linked to EBV-associated neoplasms in diverse populations, including that from Brazil.^16^ ^,^ ^18^ ^-^ ^22^ ^,^ ^24^ ^,^ ^25^ Recently, in an unprecedented analysis encompassing the entire length of the *LMP1* gene from lymphoma biopsies and saliva from asymptomatic individuals, our group described variants related to the African Raji strain in southeastern Brazil.^18^ Notably, most of the sequences that clustered closely with the Raji strain harboured shared synapomorphies and were isolated from lymphomas. Conversely, a lower frequency of this variant was detected in asymptomatic cases, which were mainly related to the Mediterranean clade. Additionally, the South American/Raji clade was highly diverse when evaluating other EBV targets compared to the Mediterranean clade. Furthermore, to our knowledge, Raji variants are the main *LMP1* variants circulating in South America.^16^ ^,^ ^18^ Although the number of available EBV sequences from different world regions has grown exponentially with the advent of hybrid capture methods,^10^ some geographic regions are still underrepresented.^10^ ^,^ ^26^ ^,^ ^29^ ^,^ ^30^ ^,^ ^31^ Moreover, despite the effort to place the South American continent in the landscape of genetic diversity of EBV^13^ ^-^ ^18^ ^,^ ^22^ ^,^ ^24^ ^,^ ^29^ ^,^ ^31^, few genomes from this region are available in public databases, including only six from Brazil.^15^

Bearing these considerations in mind, the incipient data from the EBV genomes circulating in Brazil may hamper the understanding of the EBV diversity. Here, we report a newly EBV genome from a sample previously characterised by our group as a variant related to EBV type 1 by the *EBNA3C* gene and to the African Raji strain by the *LMP1* oncogene.^18^ The novel EBV genome was characterised without the repetitive regions and was isolated primarily from a Burkitt’s lymphoma biopsy in 2005 from an individual from Rio de Janeiro, Brazil. The new genome of Brazilian EBV is herein designated CEMO3 and was further analysed with other genomes from diverse geographic area with the aim of understanding the diversity of circulating EBV in Brazil and in South America in a genomic perspective.

## MATERIALS AND METHODS

*Patient and sample characteristics* - The EBV genome was sequenced from a whole bulk tumour biopsy from a Brazilian patient with 4-year-old, female of black ethnicity with a diagnosis of HIV-associated Burkitt lymphoma (BL). The biologic sample corresponded to a retroperitoneal tumour characterised by a 46, XX, t(8;14) (q24;32) karyotype. EBV status was confirmed by EBER-specific *in situ* hybridisation of EBER-EBV. The diagnosis of BL was established by morphologic criteria according to the WHO classification.^32^ Demographic and clinical data were provided by INCA’s Department of Pathology (DIPAT-INCA). The Research Ethics Committee of INCA approved this study (CAAE approval 53571116.4.0000.5274).

*Total DNA extraction and EBV genome mapping via next-generation and Sanger sequencing* - Fresh tumour cells were isolated, and whole DNA was extracted using a QIAamp DNA Blood Mini Kit (Qiagen, Germany). DNA sequencing data of the whole tumour mass were obtained by the Illumina platform (Illumina Inc., USA) using the Nextera paired-end library in a HiSeq 2500 sequencer at INCA (200 cycles paired-end run). The quality of sequence reads was evaluated and trimmed considering a minimum length of 50 nt and quality Phred score of Q15 using the BBDuk plugin (version 35.82) in Geneious (version 9.1) software. The qualified reads were mapped to the EBV type 1 prototype (NCBI number: NC_007605) using the BBMap plugin (version 35.82) in Geneious (version 9.1) software. Additionally, polymerase chain reactions (PCRs) were designed with primers generated for this work [Supplementary data (Table)] and from the literature^33^ to cover some regions with gaps and low coverage outside the repetitive regions. Then, PCR products were purified and sequenced by Sanger using the BigDye Terminator kit (Thermo Fisher Scientific) in an automated ABI 3130xl Genetic Analyzer (Thermo Fisher Scientific). Sequences were assembled and manually edited with SeqMan v.7.0.0 (DNAStar Inc., Madison, WI).

*Genomic dataset compilation, sequence alignment and phylogenetic analysis* - A comprehensive dataset comprising 67 EBV reference genomes was compiled from publicly available databases. These genomes were carefully selected to represent a wide range of cellular lineages and geographic clades. The novel EBV genome generated in this study, obtained from paediatric patient with Burkitt lymphoma HIV+, was added to this dataset to assess its genomic diversity. The compiled dataset underwent sequence alignment using the MAFFT program.^34^ The Gblocks tool was employed to refine the sequence alignment and eliminate potential biases introduced by nonconserved and gap-rich regions.^35^ The final alignment was subjected to maximum likelihood phylogenetic analysis using the PhyML program,^36^ employing the GTR+G+I nucleotide substitution model. The aLRT-SH-like method was utilised to evaluate the branch support of the tree. The phylogenetic tree was visualised using the Figtree program (http://tree.bio.ed.uk/software/figtree/- accessed on 13 June 2023).

*Polymorphism characterisation in different EBV subgenomic regions* - The new EBV genome assembled, was classificated as EBV type 1 or 2, through the characterisation of the region of EBNA3C polymorphisms, as described in Sample et al.^37^ The EBNA-1 protein was classified as described in Gutiérrez et al.^27^ The variation in the promotor zone (Zp) and coding region of the lytic *BZLF1* gene were characterised as described by Lorenzetti et al.^14^ ^,^ ^38^ Last, the selection and evaluation of the three main polymorphisms in the *LMP1* oncogene (I124V/I152L, ins15 and del30) was due to their functional gain^39^ ^,^ ^40^ and the frequent report of these polymorphisms in South American Raji variants by the *LMP1* oncogene.^16^ ^,^ ^18^

## RESULTS

**Fig. 1:**
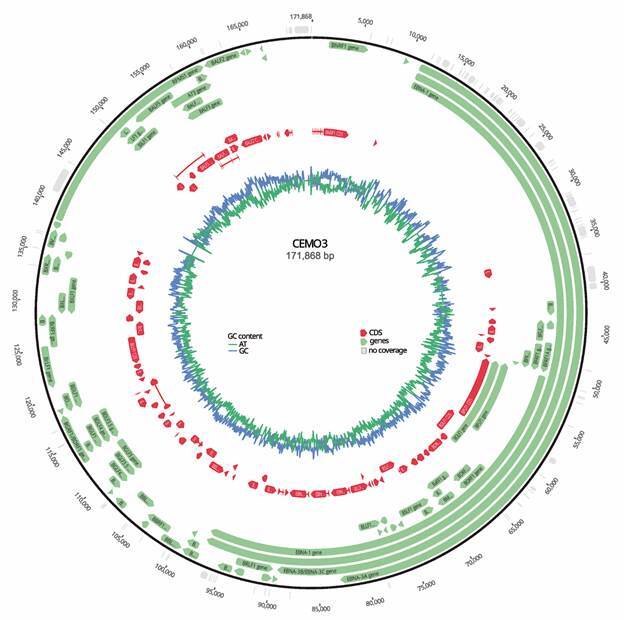
CEMO3 genome scheme. The genes are shown in green, and the coding regions (CDS) are shown in red. The GC/AT content graph is shown in blue and green, respectively. Genome annotation was performed using the Epstein-Barr virus (EBV) type 1 prototype as a reference in Geneious software. bp: base pairs; G: guanine; C: cytosine; A: adenine; T: thymine. Accession number: OQ408333.

*Characteristics of the novel Brazilian EBV near-full-length genome* - Whole-genome bulk EBV sequencing resulted in 23,217 reads after the trimming of 3,652,270 paired-end reads, with a mean length of 111 nt. An additional genomic fragment of 8,706 bp obtained by Sanger sequencing was used to fill the regions without coverage and was joined to the CEMO3 consensus genome obtained from next-generation sequencing. The final EBV genomic sequence length was 172,713 base pairs (Fig. 1), with a mean coverage of 14 bases (minimum = 0, and maximum = 106 bases). Positions with no coverage were filled with Ns. The CEMO3 genome showed 97.1% pairwise identity with the EBV prototype type 1 genome and was deposited in GenBank under accession number OQ408333.

**Fig. 2:**
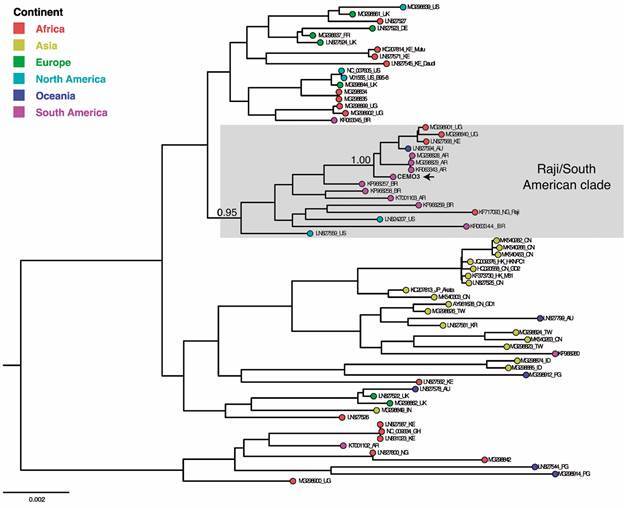
global phylogenetic reconstruction of Epstein-Barr virus (EBV) genome sequences. The maximum likelihood analysis was performed with the new CEMO3 genome and 67 other EBV genomes from GenBank, including prototypes type 1 (NC_007605) and type 2 (NC_009334), and 65 genomes representing different continents. The coloured circles on the tips represent the geographic region of the different isolates, following the legend. The new CEMO3 Brazilian EBV genome is highlighted with a black arrow. Taxon names represent the GenBank accession number, the country-of-origin abbreviation when available and the cell lineage name when applicable. US: United States; UK: United Kingdom; DE: Germany; FR: France; KE: Kenya; UG: Uganda; AU: Australia; AR: Argentina; BR: Brazil; NG: Nigeria; CN: China; HK: Hong Kong; JP: Japan; TW: Taiwan; KR: Korea; ID: Indonesia; PG: Papua New Guinea; IN: India; GH: Ghana.

*The CEMO3 strain from a Brazilian patient with Burkitt lymphoma is related to the Raji strain* - The global phylogenetic tree of EBV complete genomes showed two major clades segregating the strains related to prototypes 1 (NC_007605) and 2 (NC_009334) (Fig. 2). The prototype 2-related clade showed less geographic diversity, harbouring sequences from Africa, two from Oceania, and one from South America. The prototype 1-related clade exhibited the most geographic diversity, encompassing two subclades. One is markedly represented by Asian strains, with few sequences from Oceania, Europe, Africa, and South America. The second group included sequences from North America, including prototype 1, together with the majority of sequences from Africa and South America and to a lesser extent from Europe and Oceania. Importantly, sequences from Europe segregate in a different subclade than those from South America. The CEMO3 and most South American EBV genomes branched in the South American/Raji clade, with high support (aLRT ≥ 0.95), within the EBV prototype 1 radiation.

*South American Raji variants harbour a unique combination of polymorphisms different from the original Raji strain* - The EBV diversity evaluated in the present work, was based on important subgenomic targets of EBV that showed that the South American Raji variants possess a high diversity in different genetic regions. Herein we will use the word “haplotypes” to discuss the blocks of polymorphisms from different genetic regions of EBV. The main haplotype among the South American genomes evaluated (yellow box in Table) harboured polymorphisms absent in the original Raji strain defined at lytic and latent proteins, BZLF-1 and LMP-1, respectively. A group of haplotypes distinctly diverse at lytic targets (red square and green box) was insufficient to segregate these Raji-related variants from the Raji strain in genomic phylogenetic analysis. Notably, the EBNA-1 protein classification has remained the same as that of the Raji strain (V-leu Arg) in most South American genomes. Nevertheless, South American Raji-related variants do not have the large deletion in EBNA3C that the Raji strain harbours, and they were classified as type 1 by this target.

**TABLE High:** diversity of haplotypes identified in South American Raji variants defined by genomic phylogenetic analysis (red square, yellow and green boxes). The haplotypes and polymorphisms of the new CEMO3 Brazilian EBV genome are highlighted in bold font

			Haplotypes of South American Raji variants				
Genome name	Access number (Genbank)	Country	EBNA2/3C	Zp	BZLF-1	EBNA-1	LMP-1
B95-8	V01555	US	Type 1	V1	Zta-C	P-ala	WT/WT/ins15/WT
Raji	KF717093	NG	Type 1	V1	Zta-C	V-leu Arg	WT/WT/ins15/WT
MP	KP968258	BR	Type 1	V1	Zta-B4	V-leu*	I124V/I152L/ins15/del30
CCH	KP968257	BR	Type 1	V1	Zta-B4	V-leu Arg	I124V/I152L/ins15/del30
CEMO3	OQ408333	BR	Type 1	V1	Zta-B4	V-leu Arg	I124V/I152L/ins15/del30
CV-ARG	KR063343	AR	Type 1	V1	Zta-B4	V-leu Arg	I124V/I152L/ins15/del30
RPF	KR063344	BR	Type 1	V3	Zta-A2	V-leu	I124V/I152L/ins15/del30
SCL	KP968259	BR	Type 1	V3	Zta-A2	V-leu Arg	I124V/I152L/ins15/WT

EBV: Epstein-Barr virus; EBNA: Epstein-Barr nuclear antigen; LMP1: latent membrane protein 1; Zp: promoter of the BZLF-1 gene; WT: wild type; US: United States; NG: Nigeria; BR: Brazil; AR: Argentina; Yellow colour: EBNA3C+Zp+BZLF1+EBNA1+LMP1 haplotype; Red square: EBNA3C +Zp+BZLF1 haplotype; Green colour: EBNA3C+Zp+BZLF1 haplotype.

## DISCUSSION

The CEMO3 genome is the first assembled from a patient from Rio de Janeiro State and the seventh from Brazil deposited in a public database. The EBV CEMO3 strain genome clustered near the African Raji strain genome, confirming the previous classification based only on the *LMP1* oncogene made by our group.^18^ This EBV genome from Brazil increases the knowledge of EBV diversity at the genomic level in the Americas, since there is an incipient number of Brazilian and South American genomes deposited in public databases.^15^ To our knowledge, all EBV Brazilian genomes available in public sequence databases were obtained from Burkitt’s lymphoma patients from the São Paulo microregion. Notably, almost all of these genomes cluster within the South American/Raji clade at both the genomic and genetic (*LMP1* oncogene) levels.^18^

In our previous study,^18^ we demonstrated that the Raji and Med variants from the *LMP1* oncogene are the main EBV variants circulating in Rio de Janeiro, the second largest metropolitan region in Brazil. The South American/Raji clade included mainly primary sequences from lymphomas, while the Mediterranean clade clustered mainly sequences derived from saliva of asymptomatic individuals. Additionally, we described that variants within the South American/Raji and Mediterranean clades are the main EBV variants of the *LMP1* oncogene circulating in South America.^16^ ^,^ ^18^ Additionally, the Raji-related variants circulating in South America attracted our attention because they shelter LMP1 polymorphisms of pathological importance (I124L/I152L, ins15 and del30) significantly more frequently than the variants belonging to the Mediterranean clade. Notably, I124V/I152L represents a functional gain when compared to wild-type sequences, such as the Raji strain.^39^ The ins15 bp region encodes a signalling motif for Janus Kinase 3 (JAK3), and del30 possibly plays a role in immune escape by inducing the expression of cytokines at a lower level than other variants.^40^ Last, the variants related to the Raji strain from Brazilian samples were shown to harbour not only the Type 1 + V1 haplotype that is present in the original Raji strain but also, significantly, the Type 2 + V3 and Type 1 + V3 haplotypes when compared with the Brazilian Mediterranean variants.^18^ The potential pathogenic Type 1 + V3 haplotype is associated with diverse malignant neoplasms worldwide, and recently, this haplotype was shown to confer a functional increase in lytic reactivation due to the V3 polymorphism located in the promoter of the lytic transactivator gene *BZLF1*.^8^ All these findings indicate that the variants related to the African Raji strain can harbour unique synapomorphies, such as in the *LMP1* oncogene.^18^ In addition, high haplotype diversity and possible recombinant potential have already been suggested in previous studies.^16^ A representative sample of the South American variants related to the Raji strain described in Brazil was chosen for complete genome sequencing to contribute to knowledge of EBV diversity at the genomic level. Thus, a sample from a patient with Burkitt’s lymphoma was submitted to whole-genome sequencing by NGS. The global phylogenetic tree generated using the complete genome sequences showed two major clades, whose segregation followed the EBV prototype type 1 and type 2 classification. This segregation is expected since the classification by type is the major type of variation in the EBV genome defined almost exclusively by variation of *EBNA2* and *EBNA3* genes.^26^

Geographic restriction is the second major driver of variation detected in the EBV genome,^26^ but the understanding of the real pattern of diversity of EBV worldwide is obscured due to the absence of representative sequences of diverse geographic regions.^26^ ^,^ ^29^ ^,^ ^30^ ^,^ ^31^ Despite the few South American EBV genome sequences from the database, the present work clearly demonstrated that the new CEMO3 and most South American EBV genomes clustered closely with the African Raji strain within the South American/Raji clade with high support. All these sequences belong to EBV type 1, and the majority harbour *LMP1* polymorphisms^15^ ^,^ ^18^ absent from the original Raji strain.^41^ However, these synapomorphies are not sufficient to group the South American variants into a monophyletic clade at the genomic level. Recently, it was observed that phylogenetic analyses of different oncogenes alone can generate different phylogenetic topologies, usually paraphyletic clades that make it difficult to define clades for an adequate classification scheme.^15^ ^,^ ^30^

When several genetic targets are analysed simultaneously, the haplotype relationship of viral oncogenes is not homogeneous, raising the need for further studies to understand these different haplotype relationships from EBV genomes. The haplotypic analysis highlighted that the main changes related to the Raji strain were in the *BZLF1* and *LMP1* genes, which is expected since within their class of lytic and latent targets, respectively, they are one of the most variable genomic regions.^42^ In consonance with the literature, the diversity of lytic targets is not enough to segregate the analysed variants at the genomic level since they are less diverse than the latent targets.^26^ Alternatively, the South American EBV genomes possess other haplotypes of the Zp and BZLF-1 lytic targets, different from those in the Raji strain.^18^ ^,^ ^41^ Although not all evaluated South American genomes had all three LMP-1 oncoprotein polymorphisms evaluated, the synapomorphy I124L/I152L that characterises the South American Raji variants was present in all.

Related to the main polymorphisms of EBNA-1, this latent protein demonstrated to present the same variant protein classification of the Raji strain in most South American samples, showing less variation than the *LMP1* oncogene as expected.^42^ Last, the variants from South America do not harbour the large deletion in EBNA3C that is present in the Raji strain genome,^41^ since they were classified as type 1 by this target. This large *EBNA3C* gene deletion was probably a consequence of the Raji lineage immortalisation process or an intrinsic characteristic of the primary tumour.

The EBV haplotypes were determined in an attempt to understand the relationship of the Brazilian strains evaluated here with the Raji strain. Haplotypic investigation is necessary since the previous single-gene phylogeny and polymorphism classification may not accurately represent the true diversity of a genome-wide herpesvirus. The multigenic approach remains an important tool for understanding the virus, which can provide useful information on genetic diversity and divergence that might otherwise be obscured.

Altogether, these data confirm at the genomic level the close relationship between African and South American EBV variants. Further analyses are necessary to better understand the dispersion of EBV strains from different admixed ancestries and in which way the intrinsic genetic and ethnographic characteristics of South America allowed the adaptation and persistence of strains in this geographic region.

*Data availability* - The new EBV genome sequence CEMO3 was submitted to GenBank: OQ408333 and will be available at the time of publication of the manuscript.
