# Supplementary material for: Unique synapomorphies and high diversity in South American Raji-related Epstein-Barr virus genomes
Source: Mem Inst Oswaldo Cruz. 2023 Nov 3;118:e230122. doi: 10.1590/0074-02760230122 (PMC10629697; doi:10.1590/0074-02760230122)
Supplement: Supplementary file 1 [file 1678-8060-mioc-118-e230122-s.pdf]

TABLE

List of primers used in Sanger sequencing

Primer pair	Primer name	Sequence	Genome coordinate	Product in base pairs	Annealing temperature in Celsius
1[a]	39911	CTGGTTCTGGGGCTCCTCT	39911	221	56
			145435		
	40112	CAGGTCGGACTAGCGGATG	40112		
2[a]	40944	CACCCTTCCTCCTTCCGTTT	40944	482	54.1
	41405	AAATCACCCCAAGTCCCCTTC	41405		
3[a]	48386	CATTCAGTGGATGTGGTACCG	48386	242	52.6
	48608	TGAAACACTTGTAGGCCCGG	48608		
4[a]	63017	CGGCCCGGCGTCTAAATATT	63017	290	54.7
	63287	GAACGGGTCACTCTGCTTGA	62287		
5[a]	64555	CGGAGATTGAGAGTGACATGG	64555	290	52.1
	64971	GTGGCTGTTGATCATCCTCAT	64971		
6[a]	87439	CAGTGAAGCGCACAAATGTTA	87439	306	51
	87721	TATTTGGCTGTGACACTGACG	87721		
7[a]	88592	GTCTCAAGATCCTGTGCATGG	88803	452	52.6
	89023	CCTGTGCTTCAGCAGTAGCTT	88808		
8[a]	100708	AATTCTTTTGGAGCCAGGTGT	100708	355	51.6
	101042	TTTGGACACCTCATGAAGACC	101042		
9[a]	105498	AATTCGTAGAGCCACCAAGTG	105498	364	52
	105841	CAGATTTCCAGCCCTATGTCC	105841		
10[a]	108015	CTCCTCCGTTTTACCGATGAC	108015	426	52.2
	108421	GGTAAAGGGGGAGGCATAAC	108421		
11[a]	136417	ATGCTATCAGGTAACGCAGGA	136417	470	52.8
	136867	TCTCTTTTCCGCTTGTTGGAT	136867		
12[a]	143795	TGGGTGGAGCTAGGTAGGATT	143795	656	54.2
	144430	TGTGTACCCAGGGTGAGAGAC	144430		
13[a]	168997	CTCTCAAGGTCGTGTTCCATC	168997	679	52.2
	169655	CCTCCACTTTTTCCAGGAATG	169655		
14[b]	60125	AACAGGCGGGCGAATGTGTAAT	60125	1272	56.2
	61397	ACCTTTCATCCGAACTCCTCAGGT	61397		
15[b]	96177	TTGCATTGGCTGCAAAGGGG	96177	614	56.4
	96791	CACCACGTCCACGACCTCTC	96791		
16[b]	157382	TCAGCACCATGTTCTGCCTCTT	157382	507	55.9
	157889	TTCGTGACCAACACAACCGT	157889		
17[b]	51199	CCGAAATAGGGCCTTGCCATCAAT	51199	1046	56.8
	52245	ATTTCAGGACTACCTGCGCGACTT	52245		

a: reference: this work; *b*: reference number 33; A: adenine; C: cytosine; G: guanine; T: thymine.

CC BY
